# Comprehensive Profiling of microRNA Biomarkers for the Assessment of Male Infertility

**DOI:** 10.7759/cureus.95470

**Published:** 2025-10-26

**Authors:** Manoharan Shunmuga Sundaram, Sanjeeva Reddy, Vettriselvi Venkatesan, Madan Kalagara

**Affiliations:** 1 Reproductive Medicine, Sri Ramachandra Institute of Higher Education and Research, Chennai, IND; 2 Reproductive Medicine and Surgery, Sri Ramachandra Institute of Higher Education and Research, Chennai, IND; 3 Human Genetics, Sri Ramachandra Institute of Higher Education and Research, Chennai, IND; 4 Pathology, Vijaya Medical Centre, Vishakapatnam, IND

**Keywords:** asthenozoospermia, biomarker, male infertility, microrna, oligozoospermia, qrt-pcr, teratozoospermia

## Abstract

Introduction

Male infertility is a global health concern, and conventional semen analysis often provides limited insight into underlying molecular mechanisms. Seminal plasma microRNAs (miRNAs) have emerged as promising non-invasive biomarkers reflecting spermatogenic and epididymal function. This study investigated the expression of eight candidate miRNAs in men with asthenozoospermia, oligozoospermia, teratozoospermia, and fertile controls.

Methods

Semen samples (n=22 per group) were classified according to the WHO 2010 criteria. Total RNA was extracted from seminal plasma, and miRNA expression was quantified by real-time quantitative polymerase chain reaction (RT-qPCR) using miR-532-5p as the endogenous control. Relative expression was calculated using the ΔΔCt method. Statistical comparisons were performed using Student's t-test, and diagnostic potential was evaluated by receiver operating characteristic (ROC) analysis.

Results

In asthenozoospermia, miR-139-5p was significantly downregulated, showing the highest diagnostic potential (area under the curve (AUC)=0.88), while other miRNAs demonstrated minimal changes. In oligozoospermia, moderate expression alterations were observed for miR-932-5p and miR-942-5p, with fair diagnostic performance. Teratozoospermia exhibited no statistically significant miRNA differences, though some candidates showed weak-to-fair ROC-based diagnostic ability (AUC range: 0.69-0.88).

Conclusion

Seminal plasma miRNAs display phenotype-specific expression patterns and potential as non-invasive biomarkers for male infertility. miR-139-5p appears most promising for asthenozoospermia, while miR-932-5p and miR-942-5p may have relevance in oligozoospermia. These preliminary findings, derived from a modest sample size and exploratory design, warrant validation in independent, larger cohorts to confirm reproducibility and clinical utility.

## Introduction

Male infertility is a major global health concern, affecting approximately 15-20% of couples [1]. Standard semen analysis, assessing sperm concentration, motility, and morphology, has long been the primary diagnostic tool [2-4]. Deviations from reference values define conditions such as oligozoospermia, asthenozoospermia, and teratozoospermia. While essential for initial screening, semen analysis is largely descriptive and provides limited insight into the molecular or cellular causes of infertility [5]. Its predictive power is also imperfect, as some infertile men present with normal semen parameters, while others with abnormal values may conceive naturally [6,7]. These limitations highlight the need for molecular biomarkers that can enhance diagnosis and clinical management.

Spermatogenesis is a complex process transforming diploid spermatogonia stem cells into haploid spermatozoa over approximately 64 days in the seminiferous tubules [8,9]. This process is orchestrated by precise gene expression and epigenetic regulation [10-12]. Among the key epigenetic regulators are microRNAs (miRNAs), short (~22 nucleotides) non-coding RNAs that primarily control post-transcriptional gene expression [13,14]. MiRNAs regulate target mRNAs, leading to decreased protein expression [15]. Their critical role in male reproduction is increasingly evident. The disruption of miRNA biogenesis enzymes, such as Dicer and Drosha, in animal models causes severe testicular defects and cessation of sperm production [8,10,16].

Mature spermatozoa harbor a distinct and stable repertoire of miRNAs originating from spermatogenic and epididymal maturation stages. Rather than being mere developmental remnants, these miRNAs possess regulatory potential that may influence post-fertilization events. Experimental studies in murine and in vitro human models have shown that paternal miRNAs, such as miR-34c and miR-449a, are actively delivered to the oocyte during fertilization, contributing to zygotic genome activation, chromatin remodeling, and early embryonic cleavage [6,11]. Although the precise molecular mechanisms and functional significance of sperm-borne miRNAs in natural human fertilization remain incompletely characterized, their role in regulating early embryogenesis is increasingly recognized. This dual role positions sperm miRNAs as ideal biomarkers for male infertility [11,17,18]. Aberrant expression, either upregulation or downregulation, can contribute to sperm pathologies; for example, increased expression of a miRNA targeting genes essential for motility may cause asthenozoospermia, whereas decreased expression of miRNAs that suppress inhibitory genes may result in oligozoospermia [1,8,19]. Unlike subjective morphology assessments, miRNA expression can be measured with high precision using real-time quantitative polymerase chain reaction (RT-qPCR), and their stability in seminal plasma enhances their clinical utility [20-22]. Identifying specific miRNA signatures associated with sperm pathologies could therefore improve diagnostic accuracy and inform potential therapeutic strategies [15]. 

## Materials and methods

Study design, setting, and ethical approval

The entire cross-sectional study was conducted at Sri Ramachandra Institute of Higher Education and Research (SRIHER), in Chennai, India, after review and approval by the Institutional Ethics Committee (Approval No. IEC-NI/20/FEB/74/08), with all patients providing written informed consent prior to participation.

Study population

A total of 88 semen samples were collected from men aged 28-35 years attending the Department of Reproductive Medicine and Surgery, Sri Ramachandra Medical Center. Samples were classified according to the WHO 2021 semen parameter guidelines into four groups: teratozoospermia (n=20; abnormal morphology), asthenozoospermia (n=20; reduced motility), oligozoospermia (n=20; low sperm concentration), and normozoospermia (n=28; fertile controls). 

Study design considerations

The sample size (88 participants) and subdivision into four groups limit statistical power and generalizability. The study was designed as a pilot investigation to identify potential seminal miRNA candidates associated with male infertility. The age range (28-35 years) was chosen to minimize variability arising from age-related and endocrine factors, although broader age inclusion is warranted in future studies. Participants were recruited from a single tertiary center, and controls were age-matched fertile men with documented paternity. Lifestyle and environmental confounders were recorded and considered where feasible. 

Inclusion Criteria

Participants maintained three to five days of sexual abstinence prior to collection. The infertility groups included men with ≥12 months of unprotected intercourse without conception. Normozoospermic controls had proven fertility, normal WHO semen parameters, no sexual or ejaculatory dysfunction, and partners without female infertility factors.

Exclusion Criteria

Men with urogenital or systemic diseases, sexually transmitted infections (STIs), prior reproductive surgeries, recent fertility-affecting medications or hormonal therapy, unhealthy lifestyle habits, occupational or reproductive toxicant exposure, cryptorchidism or genitourinary anomalies, and improperly collected samples were excluded.

Semen purification and RNA extraction

Semen samples were collected by masturbation into sterile RNase-free containers and liquefied at room temperature (30-60 minutes). Standard parameters (volume, pH, concentration, motility, and morphology) were recorded [23-25]. Samples were diluted (1:1) with cold RNase-free Phosphate-Buffered Saline (PBS( and centrifuged (300 × g, 10 min, 4 °C). Pellets were resuspended in somatic cell lysis buffer (0.5% Triton X-100, 0.1% SDS) on ice to eliminate round cells, followed by centrifugation (800 × g, 10 min, 4 °C) and PBS washing to yield purified sperm pellets.

RNA was extracted using the TRIzol method [2,22]. After chloroform phase separation, RNA was precipitated with isopropanol, washed, air-dried (20-30 min), and resuspended in nuclease-free water (55-60 °C). RNA concentration (40-50 ng/µL) and purity (A260/A280 = 1.8-2.0) were assessed with a NanoPhotometer [26].

cDNA synthesis and miRNA quantification

RNA underwent poly-A tailing and reverse transcription using the TaqMan™ Advanced miRNA Kit, followed by pre-amplification per manufacturer’s protocol [4,27]. Amplified cDNA was diluted and used for quantitative real-time PCR (RT-qPCR). Expression of hsa-miR-139-5p, miR-34b-3p, miR-942-5p, miR-1208-5p, miR-296-5p, miR-93-3p, miR-328-3p, and miR-1260a-5p was quantified using TaqMan™ Advanced miRNA Assays on Rotorgene Q and Applied Biosystems 7900HT systems with SYBR Green chemistry with confirmation by gel electrophoresis. miR-532-5p served as the endogenous control [4,12]. Thermal cycling followed standard TaqMan conditions, with melt-curve analysis performed for specificity.

Data and statistical analysis

Cycle threshold (Ct) values were recorded for each sample. ΔCt values were calculated by subtracting Ct of the endogenous control (miR-532-5p) from each target miRNA. ΔΔCt values were obtained by subtracting the mean ΔCt of normozoospermic controls from each sample's ΔCt. Fold changes were calculated as 2^-ΔΔCt [2,27].

Group differences were analyzed by Student's t-test (p<0.05 was considered significant). Receiver operating characteristic (ROC) curve analysis (GraphPad Prism 8.0.2) was performed to evaluate the diagnostic sensitivity and specificity of each miRNA [28].

## Results

Relative expression of candidate miRNAs in different sperm abnormalities 

The expression levels of eight miRNAs (miR-139-5p, miR-34b-3p, miR-932-5p, miR-296-5p, miR-93-3p, miR-328-3p, miR-1260a-5p, and miR-1208-5p) were quantified in spermatozoa from men with teratozoospermia, asthenozoospermia, and oligozoospermia, and compared with fertile controls. Expression was normalized to internal controls and expressed as ∆Ct, ∆∆Ct, and fold change (FC) values (Table 1).

**Table 1:** Relative expression of candidate miRNAs in different sperm abnormalities miR - MicroRNA; ΔCt (Delta Ct) - difference between the cycle threshold (Ct) of the target miRNA and the reference gene (U6 snRNA); ΔΔCt (Delta–Delta Ct) - difference in ΔCt between each patient group and fertile controls. Fold change (FC) was calculated as 2⁻ΔΔCt, with FC>1 indicating upregulation and FC<1 indicating downregulation. Expression levels were measured by RT-qPCR (reverse transcription–quantitative polymerase chain reaction) using U6 snRNA as an internal reference. Statistical comparisons among teratozoospermia, asthenozoospermia, and oligozoospermia groups were performed using one-way ANOVA with Tukey's post hoc test (p<0.05 considered significant).

Relativeexpression of miRNAs	Teratozoospermia			Asthenozoospermia			Oligozoospermia		
	∆Ct	∆∆Ct	FC	∆Ct	∆∆Ct	FC	∆Ct	∆∆Ct	FC
miR-139-5p	0.594	2.389	0.191	4.423	6.217	0.013	-2.02	-0.225	1.169
miR-34b-3p	-1.662	0.093	0.937	0.615	2.37	0.193	-1.989	-0.234	1.176
miR-932-5p	1.024	1.838	0.28	0.705	1.52	0.349	1.673	2.487	0.178
miR-296-5p	-0.917	-0.127	1.092	1.689	2.48	0.179	-1.173	-0.382	1.303
miR-93-3p	1.69	-0.05	1.035	-0.15	-1.89	3.706	1.58	-0.16	1.117
miR-328-3p	1.26	-0.1	1.072	-1.35	-2.71	6.543	1.29	-0.07	1.05
miR-1260a-5p	2.07	-0.04	1.028	0.31	-1.8	3.482	0.19	-1.92	3.784
miR-1208-5p	2.05	1.18	0.441	-0.529	-1.399	2.637	2.56	1.69	0.31

Distinct expression patterns were observed across groups. miR-139-5p was markedly downregulated in teratozoospermia (FC≈0.19) and asthenozoospermia (FC≈0.01), but slightly upregulated in oligozoospermia (FC≈1.17). miR-34b-3p showed strong downregulation in asthenozoospermia (FC ≈ 0.19), mild upregulation in oligozoospermia (FC≈1.18), and minimal change in teratozoospermia. miR-932-5p was consistently reduced, most prominently in oligozoospermia (FC≈0.18).

Asthenozoospermia exhibited the most pronounced alterations, with significant suppression of miR-139-5p and strong upregulation of miR-93-3p (FC≈3.71), miR-328-3p (FC≈6.54), and miR-1260a-5p (FC≈3.48). Oligozoospermia displayed a mixed profile, with both upregulated (miR-296-5p, miR-1260a-5p) and downregulated (miR-932-5p, miR-1208-5p) miRNAs. Teratozoospermia changes were generally mild, except for miR-139-5p downregulation. 

Comparative expression and statistical significance of miRNAs in infertile and control groups

Teratozoospermia

None of the eight miRNAs differed significantly from controls in the teratozoospermia group.

Asthenozoospermia

In the asthenozoospermia group, miR-139-5p (p<0.001) and miR-328-3p (p<0.001) showed significant dysregulation. Other miRNAs trended toward change but were not significant.

Oligozoospermia

In the oligozoospermia group, miR-932-5p was significantly upregulated (p=0.020), and miR-1260a-5p was downregulated (p=0.044) (Table 2).

**Table 2:** Comparative expression and statistical significance of miRNAs in infertile and control groups Mean ± SD ΔCt (Delta Ct) - values of candidate miRNAs in infertile subgroups and fertile controls. Lower ΔCt indicates higher expression. Statistical significance was determined by one-way ANOVA with Tukey's post hoc test (p<0.05). This table highlights miRNAs showing significant up- or downregulation in teratozoospermia, asthenozoospermia, and oligozoospermia.

miRNAs	Control (mean ± SDΔCt)	Teratozoospermia		Asthenozoospermia		Oligozoospermia	
		Experimental (mean ± SD ΔCt)	p-value	Experimental (mean ± SD ΔCt)	p-value	Experimental (mean ± SD ΔCt)	p-value
miR-139-5p	-1.79±3.72	0.59±4.25	0.054	4.42±3.6	0.000	-2.02±3.46	0.839
miR-34b-3p	-1.76±3.71	-1.66±3.61	0.934	0.62±5.29	0.092	-1.99±4.71	0.856
miR-932-5p	-0.81±4.13	1.02±3.67	0.136	0.71±3.67	0.216	1.67±1.92	0.020
miR-296-5p	-0.79±4.12	-0.92±5.68	0.933	1.69±4.84	0.077	-1.17±3.6	0.752
miR-93-3p	1.74±3.94	1.69±3.05	0.962	-0.15±1.39	0.052	1.59±3.71	0.896
miR-328-3p	1.37±2.63	1.27±1.89	0.887	-1.35±0.9	0.000	1.29±1.6	0.914
miR-1260a-5p	2.12±2.72	2.07±1.88	0.952	0.71±2.73	0.096	0.19±3.34	0.044
miR-1208-5p	0.88±3.41	2.05±1.85	0.185	-0.53±3.39	0.179	2.56±2.52	0.080

Diagnostic performance of miRNAs based on ROC curve analysis

**Figure 1:**
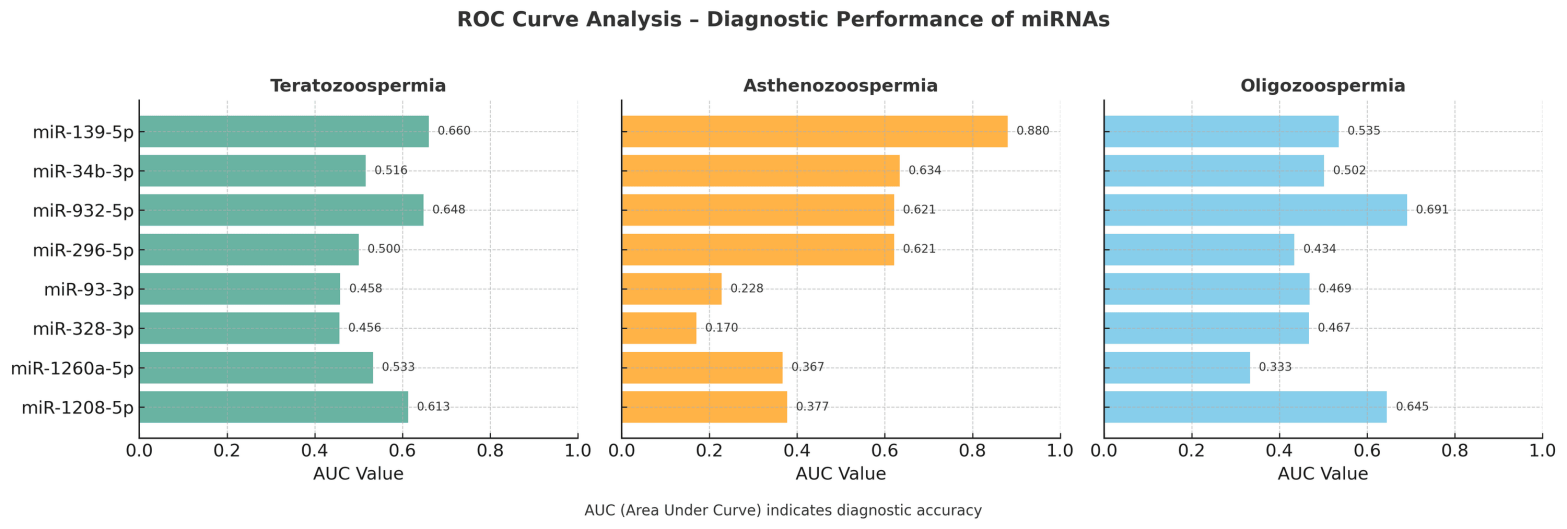
ROC curve analysis - diagnostic performance of miRNAs Diagnostic performance of miRNAs based on ROC curve analysis. Area under the curve (AUC) values indicate the diagnostic ability of each miRNA to distinguish infertile subgroups from fertile controls. Higher AUC indicates stronger discriminative power. miR-139-5p showed the highest accuracy for asthenozoospermia, while miR-932-5p and miR-1208-5p showed moderate diagnostic value for oligozoospermia. Receiver operating characteristic (ROC) analysis was based on ΔCt values obtained from real-time quantitative polymerase chain reaction (RT-qPCR).

 ROC analysis (Figure 1, Table 3) revealed phenotype-specific diagnostic potential.

**Table 3:** Diagnostic performance of miRNAs based on ROC curve analysis Area under the curve (AUC) values indicate the diagnostic ability of each miRNA to distinguish infertile subgroups from fertile controls. Higher AUC indicates stronger discriminative power. miR-139-5p showed the highest accuracy for asthenozoospermia, while miR-932-5p and miR-1208-5p showed moderate diagnostic value for oligozoospermia. Receiver operating characteristic (ROC) analysis was based on ΔCt values obtained from real-time quantitative polymerase chain reaction (RT-qPCR).

AUC values	Teratozoospermia	Asthenozoospermia	Oligozoospermia
miR-139-5p	0.660	0.880	0.535
miR-34b-3p	0.516	0.634	0.502
miR-932-5p	0.648	0.621	0.691
miR-296-5p	0.500	0.621	0.434
miR-93-3p	0.458	0.228	0.469
miR-328-3p	0.456	0.170	0.467
miR-1260a-5p	0.533	0.367	0.333
miR-1208-5p	0.613	0.377	0.645

Asthenozoospermia

In the asthenozoospermia group, miR-139-5p demonstrated excellent discrimination (AUC=0.880).

Oligozoospermia

In the oligozoospermia group, miR-932-5p (AUC = 0.691) and miR-942-5p (AUC = 0.830) showed fair to good performance.

Teratozoospermia

In the teratozoospermia group, no miRNA showed strong diagnostic accuracy; the best was miR-139-5p (AUC=0.660).

## Discussion

This study investigated the expression profiles of eight specific miRNAs in spermatozoa of men with teratozoospermia, asthenozoospermia, and oligozoospermia. The findings reveal phenotype-specific dysregulation, suggesting that seminal miRNAs can serve as promising non-invasive biomarkers for male infertility [20-22]. Unlike classical semen analysis, which is subject to biological variability, miRNA profiling may provide more objective and reproducible diagnostic information.

In asthenozoospermia, miR-139-5p was strongly downregulated while miR-328-3p was significantly upregulated. Both are implicated in flagellar function and energy metabolism essential for motility. However, diagnostic performance differed: miR-139-5p achieved excellent discriminatory power (AUC=0.880), whereas miR-328-3p showed poor accuracy (AUC=0.170) due to inter-individual variability. Thus, miR-139-5p emerges as a robust biomarker for motility disorders, consistent with earlier studies [21].

For oligozoospermia, miR-932-5p was significantly upregulated and miR-1260a-5p downregulated, implicating them in spermatogenic regulation through germ cell proliferation and differentiation [21,29]. Among these, miR-942-5p showed the best diagnostic performance (AUC=0.830), reinforcing reports linking the miR-942 family to spermatogenesis [21]. These findings highlight its potential as a reliable marker for reduced sperm counts, a critical semen parameter in fertility assessment.

In teratozoospermia, no single miRNA provided strong discriminatory accuracy (highest AUC=0.72 for miR-942-5p). This likely reflects the heterogeneous nature of morphological abnormalities arising at multiple stages of spermatogenesis. Hence, a panel-based approach combining multiple miRNAs, such as miR-942-5p and miR-1208-5p, may provide better diagnostic accuracy [30].

Phenotype-specific patterns also underline the biological roles of these miRNAs. The upregulation of miR-93-3p, miR-328-3p, and miR-1260a-5p in asthenozoospermia may represent compensatory mechanisms supporting motility by regulating protein synthesis and energy metabolism [21]. Meanwhile, miR-296-5p displayed divergent regulation, being downregulated in asthenozoospermia but upregulated in oligozoospermia and teratozoospermia, suggesting roles in sperm production and morphology [18].

Clinically, miR-139-5p and miR-942-5p showed strong diagnostic potential, with AUC values exceeding 0.80, levels comparable to established diagnostic tools in reproductive medicine. Their application could reduce reliance on repeated semen analysis and offer a more reliable assessment of motility and sperm concentration. These findings are consistent with previous literature on miRNA dysregulation in male infertility [21,23,29]. Moreover, seminal plasma miRNA profiling is non-invasive and feasible in routine clinical settings, avoiding procedures like testicular biopsy [30].

Limitations

The relatively small sample size (22 per group) limits generalizability. The pre-selected panel of eight miRNAs may not capture the full regulatory spectrum; high-throughput sequencing could reveal additional candidates. The cross-sectional design also precludes assessment of temporal changes or treatment responses. Normalization strategies and choice of endogenous controls may further influence outcomes.

## Conclusions

This study identified miR-100-5p, miR-146b-5p, and miR-942-5p as significantly downregulated in spermatozoa of infertile men compared with fertile controls. Among these, miR-942-5p demonstrated the strongest diagnostic performance, highlighting its potential as a clinically useful biomarker. These findings underscore the importance of sperm-borne miRNAs in male fertility and suggest that their profiling may complement conventional semen analysis to improve diagnostic accuracy. Further large-scale, multicenter studies incorporating functional validation are required to confirm these results and establish whether these miRNAs can be integrated into clinical practice for the assessment of idiopathic male infertility. 
